# Vitamin D status during Pregnancy and Aspects of Offspring Health

**DOI:** 10.3390/nu2030389

**Published:** 2010-03-23

**Authors:** Anne-Louise Ponsonby, Robyn M. Lucas, Sharon Lewis, Jane Halliday

**Affiliations:** 1 Murdoch Childrens Research Institute, Royal Children’s Hospital, Parkville, Melbourne, 3052 Australia; Email: sharon.lewis@mcri.edu.au (S.L.); janehalliday.h@mcri.edu.au (J.H.); 2 National Centre for Epidemiology and Population Health, The Australian National University, Canberra, 0200 Australia; Email: robyn.lucas@anu.edu.au; 3 Department of Paediatrics, University of Melbourne, Royal Children’s Hospital, Parkville, Melbourne, 3052 Australia

**Keywords:** vitamin D, ultraviolet radiation, sun exposure, pregnancy, offspring health

## Abstract

Low maternal vitamin D levels during pregnancy have been linked to various health outcomes in the offspring, ranging from periconceptional effects to diseases of adult onset. Maternal and infant cord 25(OH)D levels are highly correlated. Here, we review the available evidence for these adverse health effects. Most of the evidence has arisen from observational epidemiological studies, but randomized controlled trials are now underway. The evidence to date supports that women should be monitored and treated for vitamin D deficiency during pregnancy but optimal and upper limit serum 25(OH)D levels during pregnancy are not known.

## 1. Review

**Figure 1:**
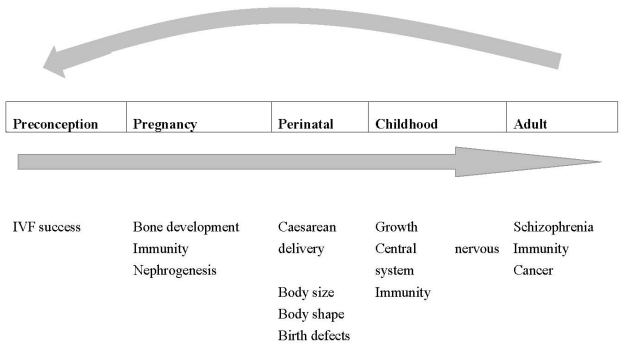
Manifestation of reviewed possible major outcomes related to vitamin D status at different life stages.

The high prevalence of vitamin D insufficiency during pregnancy is increasingly recognized, amid growing evidence that the intrauterine environment can have both immediate and long-lasting effects on health of the offspring. In this review, we outline the research evidence to date relating to vitamin D status and pregnancy, including a range of possible health outcomes from pre-conception to later life (Figure 1). With regard to terminology, we use vitamin D when referring to cholecalciferol or ergocalciferol (but ‘vitamin D_3_’ or ‘vitamin D_2_’ where reference is made specifically to only a single form of calciferol); 25(OH)D to refer to 25 hydroxycalciferol (including both 25(OH)D_2_ and 25(OH)D_3_), and 1,25(OH)_2_D, the active form of the hormone, to include both 1,25(OH)_2_D_2 _and 1,25(OH)_2_D_3_. 

## 2. Synthesis and Action of 1,25(OH)2D

Human exposure to the UV-B wavelengths of ultraviolet radiation (UVR) results in molecular instability in a cholesterol precursor (7-dehydrocholesterol) present in the skin, with the formation of previtamin D_3_. Still within the skin, previtamin D_3_ undergoes a chemical rearrangement to form vitamin D_3_ (cholecalciferol) that is taken up into the circulation largely bound to the vitamin D binding protein [1]. Vitamin D may also be consumed as part of the diet and/or in supplements [2] with the relative proportions deriving from diet or synthesis variable by location and culture. Dietary vitamin D may be in the form of vitamin D_2_ (ergocalciferol), derived from plants, or vitamin D_3_, sourced from fatty fish such as salmon, sardines and mackerel and, to a certain extent, egg yolks [3]. 

Both sunlight-derived (vitamin D_3_) or ingested (vitamin D_2_ or vitamin D_3_) vitamin D are transported to the liver where 25-hydroxylase enzymes catalyze conversion to the primary storage form (and the usual serum measure of vitamin D status), 25-hydroxyvitamin D (25(OH)D) [4]. Conversion to the active form of the hormone requires a further hydroxylation, primarily in the kidney, under the control of a 1α-hydroxylase, forming 1, 25-dihydroxyvitamin D (1,25(OH)_2_D) [4]. This step is under tight feedback control that is dependant on the body’s calcium requirements. It is now clear that a wide range of tissues possess the 1α-hydroxylase enzyme, so that local conversion of 25(OH)D to the active 1,25(OH)_2_D [5] may occur. The active hormone is best known for its role in maintaining serum calcium levels in conjunction with parathyroid hormone (PTH). 1,25(OH)_2_D increases intestinal calcium absorption [6], thereby suppressing PTH secretion [5] and promoting mineralization (and decreasing de-mineralization) of the skeleton [7]. 

### 2.1. Non-calcitropic Actions

It is now clear that 1,25(OH)_2_D also has a range of non-calcitropic functions, with locally synthesized hormone acting through both paracrine and autocrine signaling [8]. 1,25(OH)_2_D has been shown to stimulate insulin production [9] and thyroid stimulating hormone secretion [10] and improve myocardial contractility [11].

1,25(OH)_2_D is an important immunomodulator [12], potentiating antimicrobial peptide (cathelicidin) activity in human monocytes [13,14], and strengthening innate immunity. There are also effects on adaptive immune function, with attenuation of T lymphocyte proliferation and antigen specific activation [15]. Other potential immunoregulative functions are extensive and reviewed elsewhere [12].

An important role for 1,25(OH)_2_D in increasing differentiation and suppressing proliferation of monocytes was reported in 1981 [16], and was followed by work that showed that 1,25(OH)_2_D could retard cancer cell growth and improve differentiation [17]. Fetal development is perhaps the period of greatest differentiation and proliferation: any 1,25(OH)_2_D effects occurring during this time could have wide-ranging consequences. The evidence for some such effects is reviewed in later sections. 

## 3. Physiology

Extra calcium is required for fetal growth during pregnancy. The majority of this comes from the maternal diet and enhanced intestinal absorption, resulting in a total of around 25–30 g of calcium being transferred to the fetus during the pregnancy, mainly in the last trimester [18,19]. Maternal total serum calcium levels decline as the pregnancy progresses [20], but during the third trimester the fetus maintains higher serum calcium levels (reviewed in [21]) as a result of active transport of the mineral across the placenta. 

Maternal 25(OH)D levels do not vary markedly during the pregnancy unless intake or synthesis changes [22]. However, serum 1,25(OH)_2_D concentrations increase 50–100% above the non-pregnant state during the second trimester and by 100% during the third trimester [23], largely accounting for the increased intestinal absorption of dietary calcium [18]. 

25(OH)D crosses the placental barrier [19] and, at birth, cord blood 25(OH)D levels are directly correlated with maternal levels [24,25]. Fetal 1,25(OH)_2_D appears to be largely synthesized in the fetal kidney [19,20], possibly with some contribution from placenta-derived 1,25(OH)_2_D. Indeed, the placenta does contain 1α-hydroxylase [20,26], but whether this is mainly required for maintenance of adequate *local* 1,25(OH)_2_D levels in relation to immune tolerance (including successful implantation and maintenance of the pregnancy [26,27]) is not clear.

## 4. The Prevalence of Low Vitamin D Status during Pregnancy

As noted above, vitamin D adequacy depends on both endogenous, UV-induced synthesis, and exogenous sources, *i.e.,* diet and supplements. Vitamin D synthesis depends firstly on the level of ambient UV-B, with this in turn depending on latitude and altitude, time of the year and time of the day [28]. For any UV-B level, vitamin D synthesis is then dependent on the amount of skin exposed, skin pigmentation, use of sun protection such as shade, sunscreen and possibly other physical or host-related factors [29,30]. As for other population groups, pregnant women living at high latitude and low altitude, with dark skin pigmentation or skin usually covered by clothing will be at increased risk of vitamin D deficiency [31,32], unless dietary intake is high (Table 1). Furthermore, since 25(OH)D is stored in adipose tissue, there is some evidence that obesity is a further risk factor for vitamin D insufficiency.

**Table 1:** Risk factors for vitamin D deficiency.

**Low ambient UVR level **	High latitude location
	Winter season
**Inadequate sun exposure**	Built or indoor environment
	Extensive clothing cover (e.g., veiled)
	Excess sun avoidance – (shade, sunscreen)
**Host factors**	Dark skin pigmentation
	Old age
	Obesity
	Malabsorptive syndromes (e.g., inflammatory bowel disease)

Many studies have reported on the vitamin D status of pregnant women, in a range of environments (summarized in Table 2, including studies from 1997–2006). As expected, vitamin D deficiency is particularly common in darkly pigmented mothers, particularly among those who have migrated to regions with lower ambient UVR than their evolutionary heritage. Nevertheless, low vitamin D status is also prevalent in pregnant Caucasian populations, particularly those not receiving supplementation (Table 2).

**Table 2:** The prevalence of a low vitamin D status in pregnancy.

Reference	Year	Country	Population	Stage of Pregnancy	Definition	% n/N
[127]	1997	Iran	Iran women attending largest Tehran hospital	Delivery	<25 nmol/L	80% (40/50)
[128]	1997-2001	USA	African American–90% receiving prenatal vitamins	4-21 week gestation	<37.5 nmol/L	44.9% (89/194)
[128]	1997-2001	USA	White–90% receiving prenatal vitamins	4-21 week gestation	<37.5 nmol/L	2% (4/199)
[128]	1997-2001	USA	African–American	37-42 weeks	<37.5 nmol/L	29.2% (54/185)
[128]	1997-2001	USA	White	37-42 weeks	<37.5 nmol/L	5% (10/199)
[128]	1997-2001	USA	Turkish	12 week gestation	<25 nmol/L	83.5% (66/79)
[128]	1997-2001	USA	Moroccan	12 week gestation	<25 nmol/L	81.2% (56/69)
[128]	1997-2001	USA	Other non-western	12 week gestation	<25 nmol/L	59% (62/105)
[129]	1999	Ireland	Caucasians in Ulster–on supplements (54–55 ^o^N)	12 weeks	<25 nmol/L	4.5% (1/22)
[129]	1999	Ireland	No supplements	12 weeks	<25 nmol/L	44.2% (34/77)
[129]	1999	Ireland	Supplements	20 weeks	<25 nmol/L	22.7% (5/22)
[129]	1999	Ireland	No supplements	20 weeks	<25 nmol/L	50.6% (39/77)
[129]	1999	Ireland	Supplements	35 weeks	<25 nmol/L	0% (0/22)
[129]	1999	Ireland	No supplements	35 weeks	<25 nmol/L	20.8% (16/77)
[32]	1999-2000	Australia	Veiled &/or dark skinned women attending antenatal clinic	During antenatal care, when a routine blood test was ordered	<22.5 nmol/L	80.5% (66/82)
[130]	1999-2000	United Arab Emirates	Kuwait	Delivery	<25 nmol/L	40% (86/214)
[131]	2002	India	Attendees of Queen Mary’s Hosptal, Lucknow (26.8 ^o^N) Northern India – urban	Delivery–full term, live	<22.5 nmol/L	84.3% (118/140)
[131]	2002	India	Attendees of Queen Mary’s Hosptal, Lucknow (26.8 ^o^N) Northern India–urban	Rural	<56.3 nmol/L	84%(56/67)
[132]	2005-2006	India	Residents of Barabanki district, 26.8 ^o^N	Second trimester	<50 nmol/L	74.1% (103/139)

### 4.1. Vitamin D Supplementation

In most countries, the routine monitoring of serum 25(OH)D levels during pregnancy does not occur. A 2009 review has recommended that women with one or more risk factors for low serum 25(OH)D (Table 1) should be monitored at the beginning of gestation and in mid pregnancy [22]. 

A number of studies within non-pregnant adult populations have examined 25(OH)D levels in relation to different dosages, preparations or dosing intervals of oral vitamin D (reviewed in[33]). Recent recommendations suggest that the lower level of vitamin D adequacy should be a serum 25(OH)D level of 75–80 nmol/L. While there is no clear upper safe limit, a recent review suggested that the optimal 25(OH)D level was between 75–110 nmol/L and that these levels could be maintained with supplementation of 1,800 to 4,000 IU daily of vitamin D_3 _[34]. Few studies to date have examined vitamin D supplementation during pregnancy, and optimal 25(OH)D levels have not yet been defined [35,36]. A daily dose of 1000 IU/day does not appear to be sufficient to achieve and maintain vitamin D adequacy in women who are vitamin D deficient at the start of pregnancy [37,38]. Doses of over 2000 IU may be needed to achieve levels over 80 nmol/L [39,40] and RCTs are currently underway to evaluate this issue. In a recent trial of over 600 pregnant women, 400 IU and 4000 IU of vitamin D_3_daily were administered [41] and the formal publication of this report is awaited**.** Among non-pregnant women, studies have similarly shown that even high doses of vitamin D_3_ (10,000 IU/day) are not usually associated with hypercalcaemia or other toxicity [42,43,44]. A recent meta-analysis of vitamin D supplementation in relation to bone health in adults, showed that for every 1 IU increase in oral vitamin D_3_, the 25(OH)D level could be expected to increase by 0.016 nmol/L, with a relatively linear relationship at least up to 2000 IU/day [45].

## 5. Vitamin D Status during Pregnancy and the Effects on Offspring Health over the Life Course

### 5.1. Fetal Life

#### 5.1.1. Conception

Some older studies showed that vitamin D deficient rats had reduced fertility and litter size, associated with impaired ovarian function and spermatogenesis. The latter appeared to be calcium modulated and could be reversed with administration of calcium [46]. Sperm count and motility were both shown to be reduced and histological abnormalities of the testis were observed in another animal model, VDR null mutant mice [47]. However, the association between vitamin D deficiency and defective reproduction may be the result of hypocalcaemia rather than a vitamin D effect *per se*, as the infertile female VDR null mice developed normal fertility when fed a calcium rich diet [48].

In human *in vitro* fertilization (IVF) studies, women who achieved a clinical pregnancy had significantly higher follicular fluid (Fol F) levels of 25(OH)D than women with unsuccessful cycles [49]. Multiple regression analysis confirmed that Fol F 25(OH)D was associated with a successful IVF cycle, after adjusting for maternal age, body mass index, ethnicity and number of embryos transferred (p = 0.01); for each 2.5 nmol/L increase of Fol F 25(OH)D the likelihood of achieving a clinical pregnancy increased by 7% [49]. The authors suggested that higher Fol F 25(OH)D levels may enhance endometrial receptivity and implantation [49].

#### 5.1.2. Immunity

1,25(OH)_2_D has effects on immune function within both the innate and adaptive immune systems. For example, 1,25(OH)_2_D regulates the production of the antimicrobial protein cathelicidin, CAMP (LL37), an intracellular antimicrobial protein found within lysosomes of neutrophils, macrophages and also within trophoblasts [14,50]. Cathelicidin is a key element of innate immunity protecting against viral and bacterial infection. During pregnancy, a key requirement for the maternal immune system is the development of immune tolerance to the fetus. Recent work further suggests that 1,25(OH)_2_D may be important to specific aspects of placental function [51]. Within the placenta, 1,25(OH)_2_D can function as an intracrine regulator of CAMP (LL37) in trophoblasts, and may thus provide a novel mechanism for activation of innate immune responses in the placenta, assisting the pregnancy. 1,25(OH)_2_D down-regulates IL-6 production [52] and there is some evidence that adverse neurodevelopmental outcomes following maternal infections are mediated by this cytokine [53]. Ongoing research is examining the role of 1,25(OH)_2_D in decreasing the risk of infections during pregnancy and whether vitamin D status alters the risk of developing adverse outcomes following such infections. 

#### 5.1.3. Bone formation

In animal models (mainly rodents), vitamin D–deficient and –insufficient animals appear to have normal skeletal mineral content [54]. However, it is not clear that the vitamin D requirements of these largely nocturnal animals reflect human needs. Human studies are needed as inter-species variation in vitamin D requirements and metabolism may occur [55]. Among vitamin D deficient pregnant women, fetal ultrasound revealed splaying of the distal metaphysis of the fetal femur as early as 19 weeks, with the appearance being very similar to that seen in childhood (vitamin D-deficient) rickets [56]. There was a positive correlation between the maternal 25(OH)D level and the fetal metaphyseal cross-sectional area, with the latter 5% and 14% greater in fetuses of mothers who were vitamin D insufficient (serum 25(OH)D 25-50 nmol/L) and vitamin D deficient (serum 25(OH)D < 25 nmol/L) compared to vitamin D sufficient mothers (serum 25(OH)D > 50 nmol/L) [56]. There was, however, no association between maternal 25(OH)D level and fetal femur length at 19 weeks or 34 weeks gestation [56]. 

### 5.2. Perinatal Period

#### 5.2.1. Immunomodulation

Early life immunomodulation is likely to also be affected by vitamin D status. At birth, cord serum 25(OH)D levels are directly correlated with levels of IL-10, an immunosuppressive cytokine [57]. *In vitro* studies show that 1,25(OH)_2_D acts on naïve T cells to retard their development into either T helper 1 or 2 cells [58]. 

#### 5.2.2. Mode of Birth

Recent data suggest an association between vitamin D deficiency and caesarean section. In a cross sectional study, caesarean section was almost four-times more common in women with vitamin D insufficiency (serum 25(OH)D < 37.5 nmol/L) compared to vitamin D-sufficient women (serum 25(OH)D ≥ 37.5 nmol/L), after adjustment for race, age, education level, insurance status, and alcohol use (Adjusted OR 3.84; 95% CI 1.71–8.62) [59].

#### 5.2.3. Birth Weight

Observational studies of vitamin D status during pregnancy and physical characteristics of the offspring are few and provide conflicting results. One study found pregnancies of mothers with low vitamin D status were shorter (by 0.7 week; 95% CI −1.3, −0.1) [60], and the babies had poorer intrauterine long bone growth [60], while in other studies, offspring had lower birth weight [61,62,63]. However another study found the opposite, with babies of vitamin D deficient mothers being both heavier and longer, compared to the offspring of vitamin D-sufficient mothers [64]. Further examination of these findings suggests that the effects may vary according to the VDR genotype of the offspring. Thus within the offspring of vitamin D deficient mothers, there was lower birth weight in those having the FF or Ff genotype (where the F allele is associated with increased vitamin D receptor activity [65]), but not in offspring with the ff genotype (*P*-value for interaction after adjustment for potential confounding factors = 0.02) [66]. A large randomized controlled trial on this issue has been completed in South Carolina, USA, with recent presentation at the 14th International Vitamin D meeting, Brugges, Belgium, in October 2009 [67]. The media reported on a reduction in both preterm birth and small-for-dates babies, with vitamin D supplementation [41], but formal publication of the findings had not occurred at the time of writing this review, thus these reports cannot be formally evaluated. 

#### 5.2.4. Birth Defects

At this stage, all findings arise from animal studies, primarily in rats, and it is not clear whether there is a direct translation to human fetal outcomes. However, there is evidence that 1,25(OH)_2_D regulates cell differentiation and proliferation so that effects on organ development during fetal life are plausible and warrant further investigation. In rats, maternal vitamin D deficiency was associated with an increase in nephron number, but lower renal corpuscle size [68]. Low maternal vitamin D status may slow neonatal cardiac development [69] and alter brain morphology [70,71], with changes in the latter persisting into adulthood [72].

Excess vitamin D administration during pregnancy could also potentially be of concern. Rats receiving very high-dose vitamin D during gestation and early development (considerably higher than would be administered to humans) had adverse changes in elastin content and organization in the aorta consistent with increased later risk of hypertension or aneurysm [73]. Nevertheless, in two studies in pigs, vitamin D administration to two sows at doses that achieved serum 25(OH)D levels, approximating recommended human levels, was associated with coronary lesions in offspring at six weeks [74,75]. The relevance of these findings to humans remains to be determined. In 2008, a cohort study investigating these issues reported no statistically significant associations between antenatal maternal 25(OH)D levels and cardiac measures in offspring at nine years, including in blood pressure, carotid intima-media thickness, arterial compliance and cardiac structure [62].

### 5.3. Childhood and Adulthood

#### 5.3.1. Bone Health

Maternal vitamin D status influences bone health in the offspring [76]. In a UK cohort study, winter-born babies had lower bone mineral content (BMC) than those born in summer [77] and maternal serum 25(OH)D in late pregnancy was directly associated with total and lumbar spine BMC in offspring at nine years of age [77,78]. 

#### 5.3.2. Postnatal Infection

An increased risk of acute lower respiratory tract infection (ALRI) in newborn infants was associated with lower maternal 25(OH)D levels in a case-control study in Turkey [79], but there was no association between postnatal serum 25(OH)D levels and infant ALRI in a case-control study in Canadian children aged 1–25 months [80]. In a prospective study in Tanzania, children born to mothers with lower 25(OH)D levels at 12–27 weeks gestation, had a 46% (95% CI 11%–91%) increased risk of contracting HIV infection by 24 months of age, including postnatally during breastfeeding, and a 61% higher risk of dying (95% CI 25%–107%) [81].

#### 5.3.3. Immune diseases

Increased risk for a winter season-of-birth is seen in some autoimmune disorders, such as Crohn’s disease [82] and type 1 diabetes [83,84,85], although the latter finding may be limited to comparisons within homogeneous populations [86], or in those with specific susceptibility genotypes [87]. 

The Diabetes Autoimmunity Study in the Young (DAISY) cohort has observed that maternal intake of vitamin D via food, recorded at birth, was significantly associated with a decreased risk of islet autoimmunity in offspring during the first four years of life [88]. A Norwegian case control study found cod liver oil use during pregnancy was associated with lower risk of type 1 diabetes in the offspring, indicating vitamin D or the n-3 fatty acids eicosapentaenoic acid and docosahexaenoic acid in the cod liver oil, or both, may have a protective effect against type I diabetes [89]. These findings are also consistent with the finding of an inverse association between postnatal vitamin D supplementation in early life and type 1 diabetes risk [90,91].

Low vitamin D intake during pregnancy has been associated with an increased risk of asthma, eczema or hay fever [92,93], while winter birth was associated with higher IgE levels and lower IL-10 than summer birth [57]. As IL-10 is important in the development of tolerance to exogenous antigens and inhibition of mast cell degranulation [57], these findings are consistent with the notion of low vitamin D levels at birth increasing the risk of an allergic propensity. However, the ecological patterns are not consistent with this because some studies show a higher prevalence of asthma in warmer climates with higher ambient UVR than other locations [94,95,96,97]. These regional patterns are not necessarily conflicting, because they may also reflect uncontrolled confounding, in that higher house dust mite allergen levels may occur in warmer climates. A birth cohort has reported an increase in eczema and asthma among children whose mothers had higher 25(OH)D levels during late pregnancy (>75 *versus* <30 nmol/L) [62]. Randomized controlled trial data are required but not yet available.

#### 5.3.4. Central nervous system disorders

Vitamin D is a potent inducer of nerve growth factor synthesis [98]. Genes of the vitamin D pathway (25 hydroxylase, 1α hydroxylase and 24 hydroxylase) and VDR are expressed in rat brain cells [99] and the distribution of VDR and 1α hydroxylase is thought to be similar in rat and human brains [100]. In experimental studies, rats deprived of vitamin D prenatally had enlarged lateral ventricles, decreased cortical thickness and heavier and longer brains, and altered patterns of apoptosis [70,71]. 

Seasonal and population level patterns of schizophrenia incidence indicate the possible role of low intrauterine vitamin D in increasing later schizophrenia risk. This is a difficult but important area to research [101,102,103,104], with animal work indicting transient prenatal vitamin D deficiency is associated with subtle alterations in learning and memory functions in adult rats [105]. In a Finnish birth cohort study, regular vitamin D supplementation (maternal self-report) during the first year of offspring life was associated with a reduced risk of schizophrenia in males (but not females) (RR = 0.08, 95% CI 0.01–0.95) [106]. 

Observational studies provide indirect evidence that low vitamin D status during early life may increase the risk of multiple sclerosis [107,108]. These findings include a striking season-of-birth pattern in a large cohort of Northern Hemisphere MS cases [109], a congruent pattern in the Southern hemisphere [110], and a maternal parent-of-origin effect [111,112]. Using prenatal ambient UVR levels as an instrumental variable for vitamin D levels in pregnancy [113], an inverse association between maternal ambient UVR exposure during the late first trimester and MS risk was observed [110]. This inverse association was independent of region of birth, indicating that the first trimester ambient UVR level was not acting merely as a marker of longer term UVR exposure due to region of residence [110]. 

#### 5.3.5. Other candidate diseases that require further research with regard to the potential role of prenatal vitamin D status.

Seasonal patterns of birth in adults [114] and children [115] with brain tumors and epilepsy [116] have been noted and could be evidence of an effect of low vitamin D status, but this is far from conclusive. A tentative link between vitamin D status and autism has been postulated, arising mainly from the findings of population level studies [117,118]. Further, adult body mass index and obesity prevalence has been noted to vary as a function of month of birth [119,120]. In adult ecological and observational studies there is considerable evidence that vitamin D status may be related to risk or prognosis of a number of cancers, including of the prostate [121] and breast [122]. One animal study has shown that maternal antenatal vitamin D supplementation was associated with greater mean prostatic weight and a histologically more differentiated prostatic architecture in offspring in adulthood [123]. Whether such changes also occur in humans and have relevance for later prostate cancer risk is unknown. 

## 6. Overview

In summary, we have reviewed the existing evidence for a range of possible adverse offspring health outcomes resulting from low maternal vitamin D status in pregnancy. At this point in time, the evidence is largely based on studies of observational rather than experimental design. Insufficient trials have occurred for vitamin D supplementation in relation to specific clinical outcomes. Even with these trials (mostly of high risk women, many years ago) being systematically reviewed, it appears that there is not enough evidence to evaluate the effects of vitamin D supplementation in pregnancy [124]. The findings from recent randomized controlled trials [67] are eagerly awaited. The merit of achieving a relatively high vitamin D status (>100 nmol/L) has not been fully established, particularly in relation to offspring cardiovascular or atopic disease development. However, the majority of potential adverse offspring health outcomes pertain to low prenatal vitamin D status and the evolving evidence has provided greater impetus for public health efforts to ensure mothers are not frankly vitamin D deficient (<25 nmol/L) in pregnancy, although the value of treating relative vitamin D insufficiency in pregnancy is not clearly established. Women at risk of vitamin D deficiency should be monitored and treated during pregnancy for vitamin D deficiency [22].

## 7. Future Directions

For maternal health outcomes in pregnancy and common offspring outcomes that can be measured at the fetal, perinatal or infant stage, randomized controlled trials are feasible. Such trials need to carefully consider the safety profile of the interventional vitamin D administration provided. Care is also needed at the trial design and implementation stages to reduce the possibility of potential contamination of the placebo or lower dose vitamin D group by additional subject-initiated vitamin D supplementation or increased UVR exposure. The randomized controlled trial design is the best design to exclude that the range of possible health outcomes do not reflect other broad exposures such as lower socioeconomic status or nutritional deficiency.

To examine the potential long term effects of low vitamin D on offspring health, randomized controlled trials studies may not be feasible, particularly for rare diseases with later onset such as schizophrenia. For these studies, observational studies will need to include seasonal variation in vitamin D status, potentially confounding by other seasonal factors such as infection, and also deseasonalized interpersonal variation in ingested vitamin D or UVR-derived vitamin D. With regard to these deaseasonalized markers of vitamin D status, it is highly likely that vitamin D status in pregnancy will be correlated with ingested or UVR-derived vitamin D exposure for mothers preceding pregnancy and offspring after pregnancy. In this situation, the timing of low vitamin D’s effect over the life course will have to be carefully determined, using specific life course analytic techniques [125], to assess whether any low prenatal vitamin D status is independently associated with health outcomes independently of the mother’s vitamin D status before pregnancy and the offspring’s vitamin D status during postnatal life. One advance that will assist this is the recent development of a sensitive liquid chromatography - tandem mass spectrometry assay of 25(OH)D that can be used for archived neonatal dried blood spots meaning that such samples will become a useful tissue repository for testing a range of hypotheses linking developmental hypovitaminosis D and adverse health outcomes in later life [126].
